# An ‘eFP‐Seq Browser’ for visualizing and exploring RNA sequencing data

**DOI:** 10.1111/tpj.14468

**Published:** 2019-08-23

**Authors:** Alexander Sullivan, Priyank K. Purohit, Nowlan H. Freese, Asher Pasha, Eddi Esteban, Jamie Waese, Alison Wu, Michelle Chen, Chih Y. Chin, Richard Song, Sneha R. Watharkar, Agnes P. Chan, Vivek Krishnakumar, Matthew W. Vaughn, Chris Town, Ann E. Loraine, Nicholas J. Provart

**Affiliations:** ^1^ Department of Cell and Systems Biology/CAGEF University of Toronto Toronto ON Canada; ^2^ University of North Carolina at Charlotte Charlotte NC USA; ^3^ J. Craig Venter Institute Rockville MD USA; ^4^ Texas Advanced Computing Center University of Texas at Austin Austin TX USA

**Keywords:** *Arabidopsis thaliana*, data visualization, plant growth, RNA processing, RNA‐seq, temperature stress

## Abstract

**Summary:**

Improvements in next‐generation sequencing technologies have resulted in dramatically reduced sequencing costs. This has led to an explosion of ‘‐seq’‐based methods, of which RNA sequencing (RNA‐seq) for generating transcriptomic data is the most popular. By analysing global patterns of gene expression in organs/tissues/cells of interest or in response to chemical or environmental perturbations, researchers can better understand an organism's biology. Tools designed to work with large RNA‐seq data sets enable analyses and visualizations to help generate hypotheses about a gene's function. We present here a user‐friendly RNA‐seq data exploration tool, called the ‘eFP‐Seq Browser’, that shows the read map coverage of a gene of interest in each of the samples along with ‘electronic fluorescent pictographic’ (eFP) images that serve as visual representations of expression levels. The tool also summarizes the details of each RNA‐seq experiment, providing links to archival databases and publications. It automatically computes the reads per kilobase per million reads mapped expression‐level summaries and point biserial correlation scores to sort the samples based on a gene's expression level or by how dissimilar the read map profile is from a gene splice variant, to quickly identify samples with the strongest expression level or where alternative splicing might be occurring. Links to the Integrated Genome Browser desktop visualization tool allow researchers to visualize and explore the details of RNA‐seq alignments summarized in eFP‐Seq Browser as coverage graphs. We present four cases of use of the eFP‐Seq Browser for *ABI3*,*SR34*,*SR45a* and *U2AF65B*, where we examine expression levels and identify alternative splicing. The URL for the browser is https://bar.utoronto.ca/eFP-Seq_Browser/.

**Open research badges:**

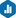

This article has earned an Open Data Badge for making publicly available the digitally‐shareable data necessary to reproduce the reported results. Tool is at http://sps:urlprefix::https; RNA‐seq data at http://sps:urlprefix::https and http://sps:urlprefix::https. Code is available at http://sps:urlprefix::https

## Significance Statement

We present a tool, the eFP‐Seq Browser, for rapidly identifying RNA sequencing samples with strong expression levels of a given gene, or where the read maps for a given gene/sample best match a particular gene model. Details can be called up with convenient links to the Integrated Genome Browser.

## Introduction

After sequence reads from an RNA sequencing (RNA‐seq) experiment are mapped to a *de novo* transcriptome or reference genome, for example the TAIR10 (Lamesch *et al*., 2012) or Araport 11 (Cheng *et al*., 2017) versions of the *Arabidopsis thaliana* genome, the resulting SAM (sequence alignment/map) or BAM (binary alignment/map) files may be explored with genome browsers that display sequence alignments in multiple tracks, typically one per library or sample type. Well‐known examples include published visualization tools such as the Integrated Genome Browser (IGB) (Nicol *et al*., 2009; Freese *et al*., 2016), JBrowse (Skinner *et al*., 2009) and others. Such tools, however, are typically designed to support visualization of a given data set after it has been identified by other means; they currently have limited ability to support a search, display summary statistics about a data set, or provide details about how an experiment was performed. Moreover, they are designed for researchers to view and assess fine‐scale details of alignments. This is useful, but researchers also need higher‐level views that let them compare data sets with each other, or sort data sets based on various criteria. In the case of RNA‐seq data, the ability to sort data sets based on expression level or congruency with specific splice variants would be particularly useful, as expression level and differential expression of splice forms play a regulatory role in many biological processes.

In order to address these shortcomings and to visualize organism‐wide gene expression patterns, we developed the eFP‐Seq Browser, a web‐based visual analytics tool that displays gene splice variants, RNA‐seq mapping coverage data, similarity of the coverage profile to the selected canonical gene model, summarized gene expression levels as colour‐coded pictographs and experimental details all in an easily sortable/searchable table. To demonstrate the potential of the eFP‐Seq Browser we developed an eFP‐Seq Browser example for the reference plant species *Arabidopsis thaliana*, focusing on two large publicly available RNA‐seq compendia and an up‐to‐date release of Arabidopsis gene model annotations designated ‘Araport11’. The two compendia include 113 RNA‐seq experiments used by Cheng *et al*. (2017) to reannotate the *A. thaliana* reference genome for the Araport11 build, and 170 RNA‐seq experiments across 69 tissues from Klepikova *et al*.'s (2016) high‐resolution map of the Arabidopsis developmental transcriptome. Data were obtained from public repositories, aligned to the reference genome assembly and then hosted on the ‘cloud’ using Amazon Web Services (AWS) S3 ‘buckets’ for low‐cost, web‐accessible storage. Data are then extracted for display in the eFP‐Seq Browser using an application programming interface (API) program running a custom compiled version of samtools (Li *et al*., 2009) on the BAR server (see Experimental Procedures). The extracted data are analysed and displayed to researchers via a browser‐based front‐end web application. Researchers can then ‘drill down’ to view alignments in the Integrated Genome Browser, which accesses the same data sets as the eFP‐Seq Browser. This open source, customizable and extensible tool will allow researchers to visualize and make unbiased comparisons of a gene's expression level and read map coverage along a selected gene model throughout the various tissues of an organism. We illustrate the utility of the eFP‐Seq Browser for identifying and exploring expression levels and alternative splicing events with four biological examples: *SR34*,* SR45a*,* U2AF65B* and *ABI3*.

## Results

### The eFP‐Seq Browser

**Figure 1:**
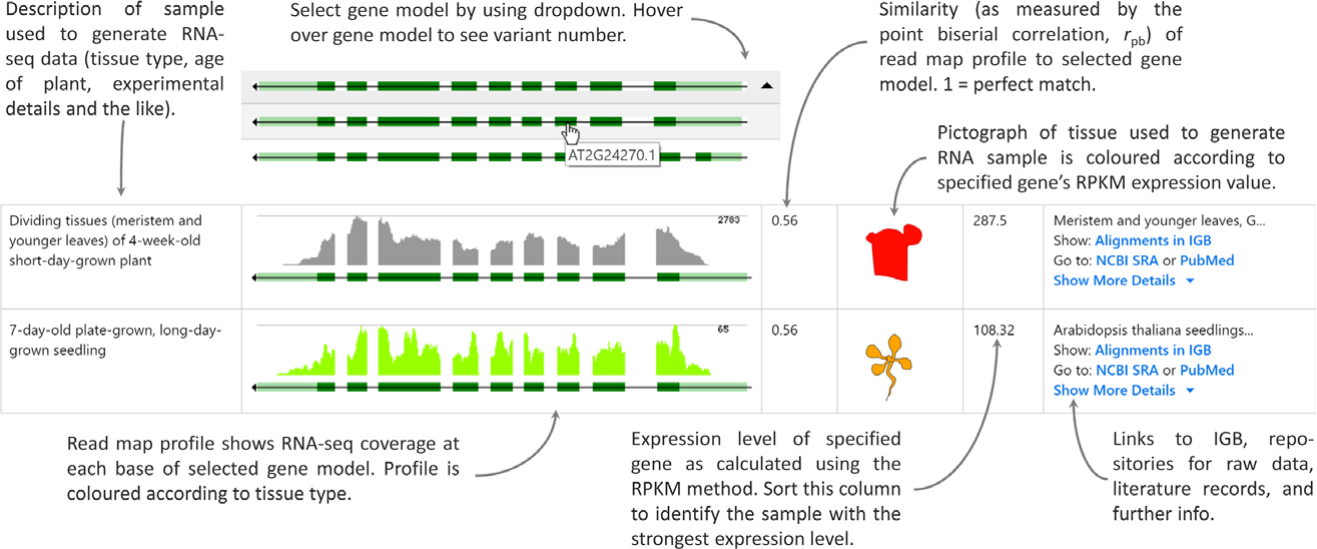
Columnar output for two samples in the eFP‐Seq Browser, with features of each column highlighted.

The eFP‐Seq Browser interface displays gene splice variants, mapping coverage of the gene of interest in all RNA‐seq experiments in a given compendium and eFP images for visual depiction of gene expression levels (Winter *et al*., 2007). Furthermore, the interface permits sorting and filtering of the records displayed based on the expression levels [as determined by reads per kilobase per million reads mapped (RPKM) values], on how well they correlate to a specific splice variant (as determined by the point biserial correlation values) or if they contain a desired keyword. This makes it a powerful visualization tool for exploring RNA‐seq data (see Figure 1 for some features).

### Using the eFP‐Seq Browser to correlate changes in splice variant abundance with developmental stage and tissue type

To demonstrate the utility the eFP‐Seq Browser we have created an example workflow for visually correlating changes in splice variant abundance with developmental stage and tissue type. We chose the serine/arginine‐rich (*SR*) genes, as their mRNAs are alternatively spliced depending on developmental stage or tissue. We then compared the visual results from the eFP‐Seq Browser with previously published *SR* mRNA splice variant quantification (Palusa *et al*., 2007).

**Figure 2:**
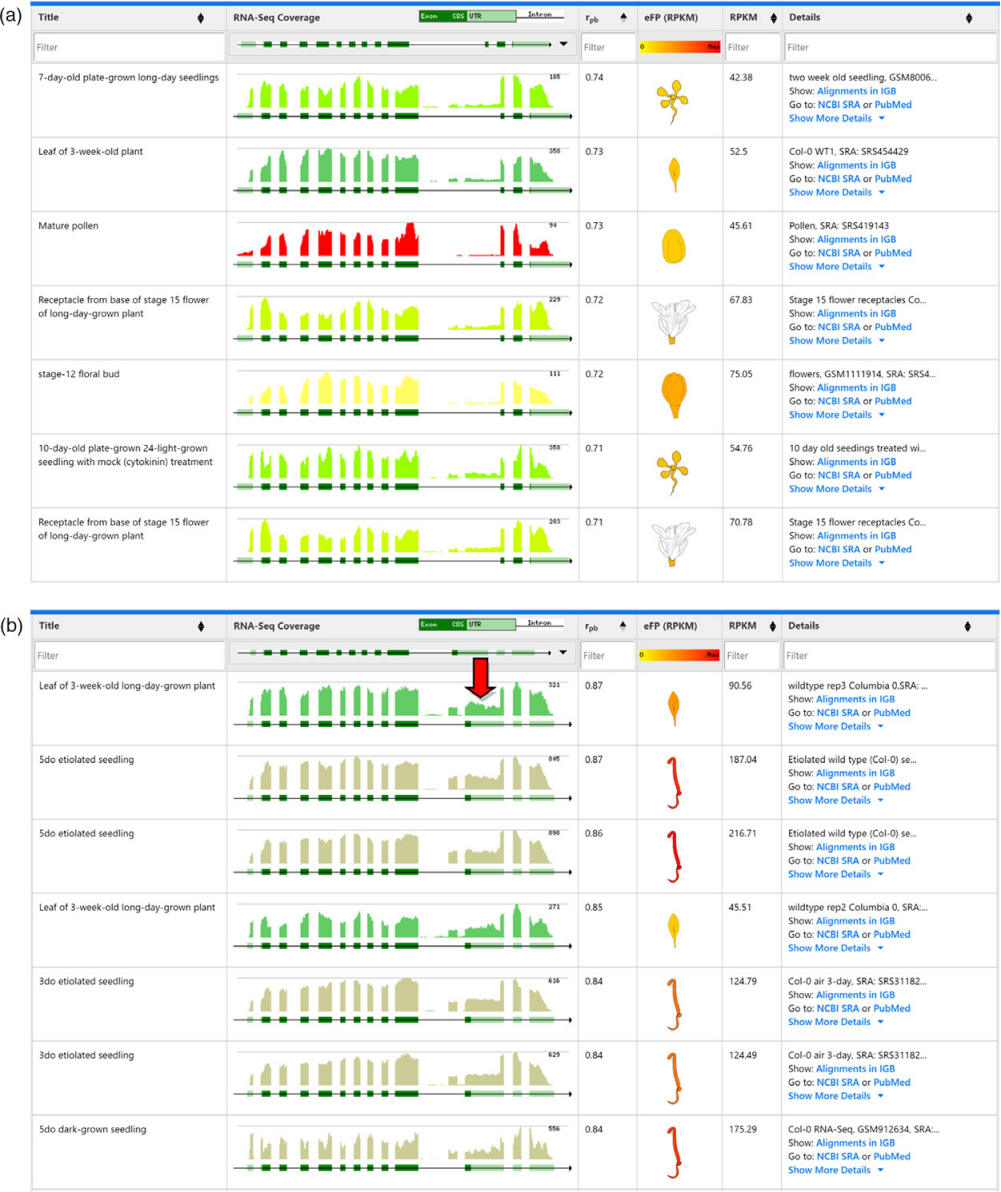
Comparison of splice variants 1 and 2 for *SR34* (*At1g02840*) in the eFP‐Seq Browser in the Araport11 data set, ordered by correlation from high to low. (a) eFP‐Seq Browser results for *At1g02840.1* (splice variant 1). (b) eFP‐Seq Browser results for *At1g02840.2* (splice variant 2). Red arrow indicates reads supportive of the alternative acceptor site in splice variant 2. Only the top seven rows are shown in each panel. 5do, 5‐day‐old; 3do, 3‐day‐old.

*SR34* (*At1g02840*) was visualized in the eFP‐Seq Browser using the Araport11 data set with the RPKM value set to the absolute scale. With splice variant 1 selected, we organized samples from top to bottom by decreasing correlation values (Figure 2a). The samples with the highest correlation values encompassed a range of tissues and stages, with a trend towards more mature stages including pollen and flower parts of older plants and leaves of 3‐week‐old plants. However, visually scanning through all the samples there were a number of reads indicating an alternative acceptor site that more closely matched splice variant 2.

Switching to splice variant 2 in the dropdown menu and then arranging by the highest correlation values strikingly changed the top displayed hits (Figure 2b). Samples from 3‐ or 5‐day‐old seedlings were now among the top hits. This visually indicates to the researcher that splice variant 2 decreases relative to splice variant 1 as the seedling develops. This is supported by Palusa *et al*. (2007), where splice variant 2 decreased in plants as they matured from day 3 to day 15. We point out that alternative splicing events can be identified by a number of different methods, including older expressed sequence tag‐based methods such as the Program to Assemble Spliced Alignments (PASA; Haas *et al*., 2003) or more recent RNA‐seq‐based methods such as cufflinks (Trapnell *et al*., 2010). For the complete reannotation of the Araport11 genome using 113 published RNA‐seq data sets (Cheng *et al*., 2017), transcriptomes for 11 different tissues were assembled using Trinity (Grabherr *et al*., 2011) followed by filtering using PASA (using the *de novo* assembled Trinity‐based transcripts as input) to confirm existing transcript assemblies or identify new ones. These gene isoforms are the ones visible in the isoform dropdown menu of the eFP‐Seq Browser. The utility of the eFP‐Seq Browser lies in being able to rapidly sort or filter RNA‐seq coverage profiles to identify samples where the greatest abundance of a given isoform occurs.

### Details‐on‐demand using links to the Integrated Genome Browser

**Figure 3:**
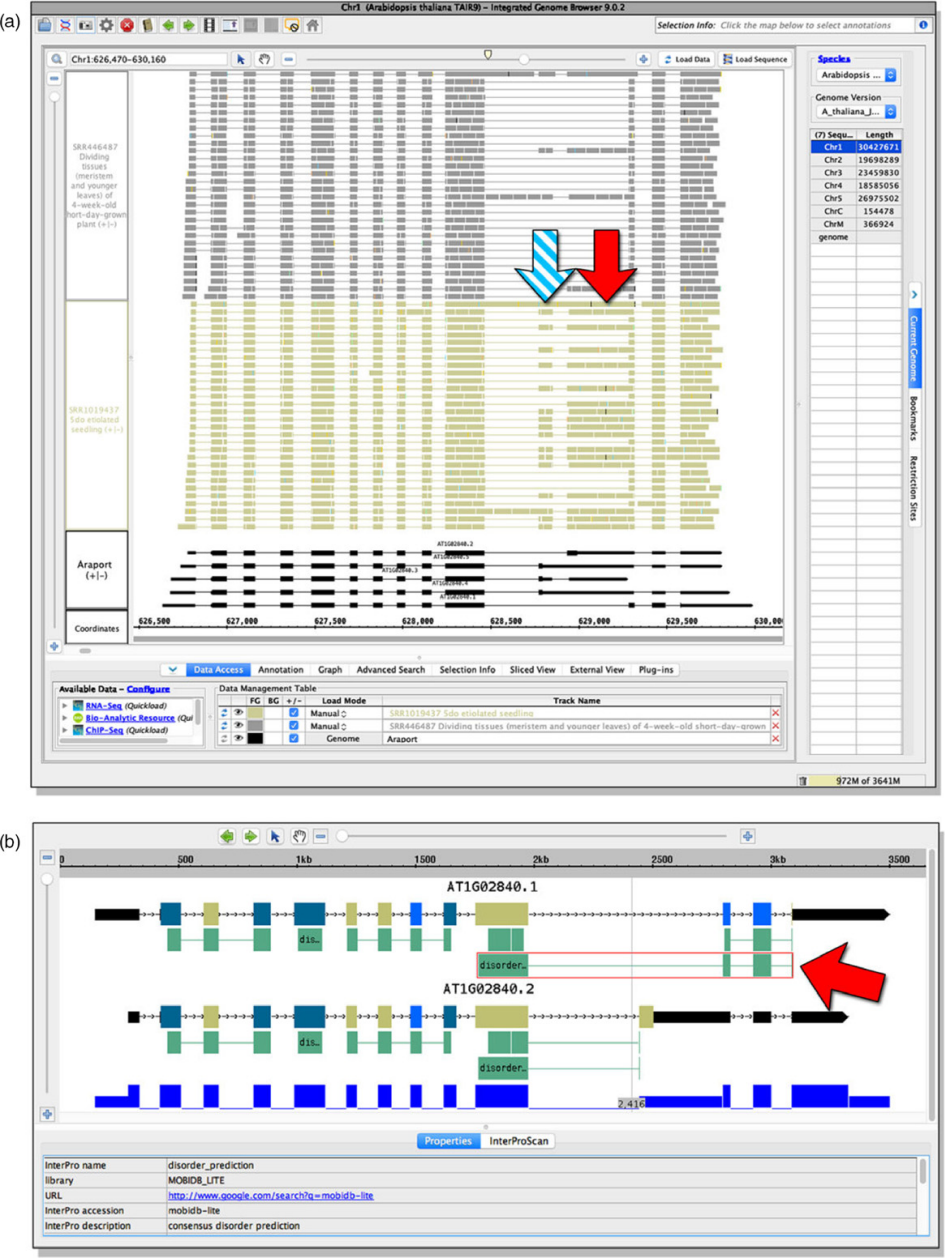
*SR34* reads in dividing tissues (meristem and younger leaves) of 4‐week‐old short‐day‐grown plants and 5‐day‐old etiolated seedlings viewed with the Integrated Genome Browser (IGB). (a) Dividing tissues and etiolated seedling sample reads visualized within the IGB main window. The solid red arrow highlights the reads within the etiolated seedling sample supportive of the alternative acceptor site for splice variant 2. The striped blue arrow highlights reads supporting an exon annotated as being in splice variant 3. (b) The IGB ProtAnnot plug‐in app comparing results from HMMPfam and MobiDB‐lite for two *SR34* splice variants, *At1g02840.1* and *At1g02840.2*. The red arrow highlights the differing lengths of predicted disordered regions in the two splice variants.

Once researchers have identified a sample of interest, they can delve further into the data by visualizing them with IGB at base‐pair resolution. In this example, a researcher may want to see how convincing the reads are for the alternative acceptor site matching splice variant 2 of the *SR34* transcript. We have compared dividing tissues (meristem and younger leaves) of 4‐week‐old short‐day‐grown plants and 5‐day‐old etiolated seedlings. In IGB, the samples are colour‐coded as they are in the eFP‐Seq Browser. Examining the two samples in IGB, the 5‐day‐old etiolated seedling appears to have many more reads supporting the splice variant 2 alternative acceptor site in *SR34* (Figure 3a). Interestingly, the 5‐day‐old etiolated seedling also has reads supporting an additional exon in *SR34* that is found within splice variant 3. This would suggest either the expression of splice variant 3 in addition to splice variants 1 and 2 or that the current annotation may be inaccurate.

In addition to the visualization of sequence data, IGB features built‐in tools for further analysis. We used the IGB ProtAnnot plug‐in (Mall *et al*., 2016) to analyse differences in predicted functional motifs between *SR34* splice variants 1 and 2. ProtAnnot shows protein annotations in the context of genomic sequence by searching InterPro and displaying protein annotations alongside gene models. Splice variant 2 has an alternative acceptor site that leads to a premature stop codon. Analysis of splice variants 1 and 2 in ProtAnnot identified both variants as having two predicted RRM domains as determined by HMMPfam (Figure 3b). However, MobiDB‐lite predicted a truncated intrinsic disordered region in splice variant 2 due to the premature stop codon. These results demonstrate how the eFP‐Seq Browser and IGB can be used to identify stage‐ and tissue‐specific splice variation through correlation ordering and visual analysis.

### Filtering in the eFP‐Seq Browser to identify stress‐related alternative splicing

**Figure 4:**
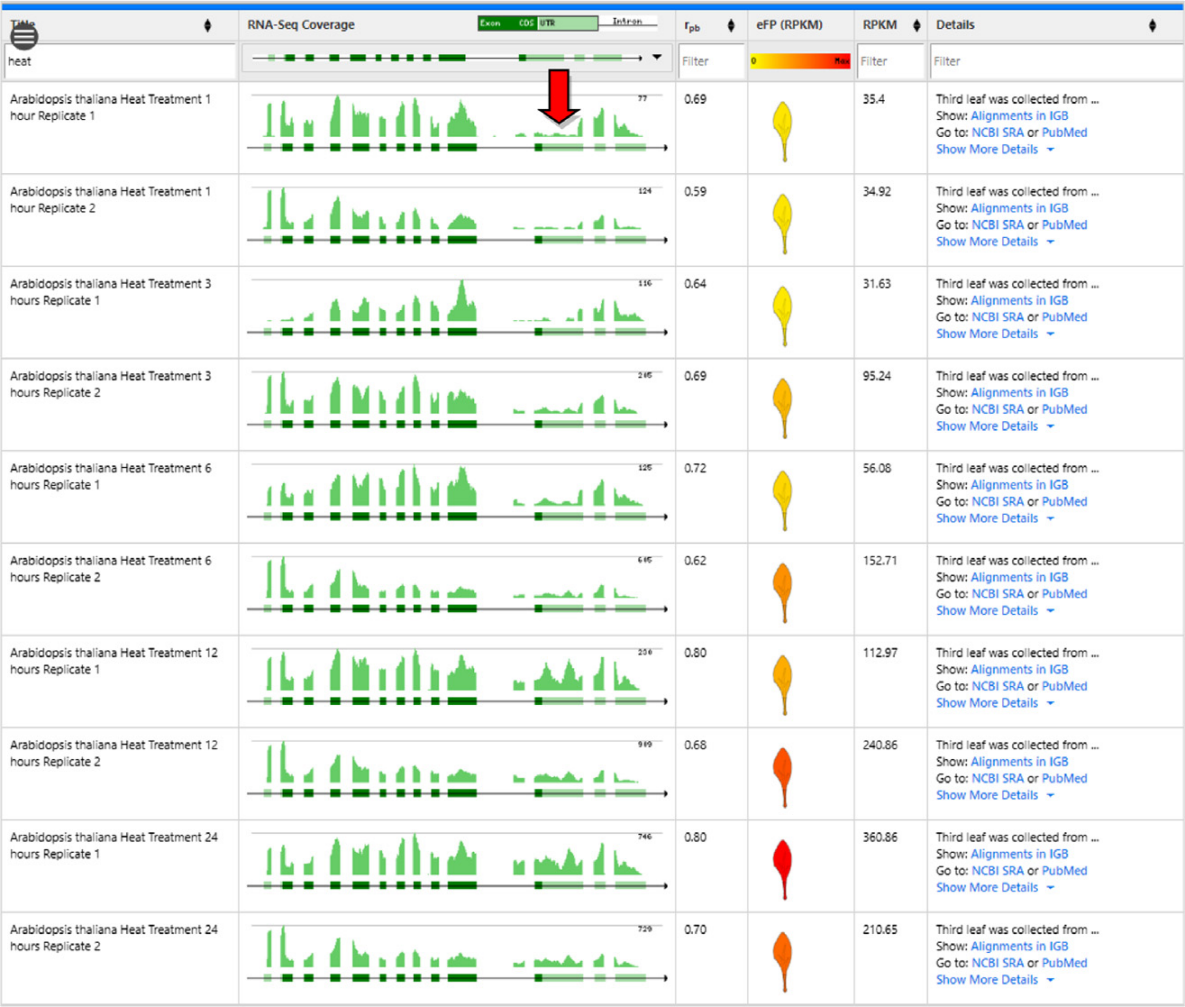
Change in splice variant abundance for *SR34* (*At1g02840.2*) following a heat treatment time course from Klepikova *et al*. (2016). The red arrow indicates the retained intron of splice variant 2. Earlier timepoints are at the top of the table, while later timepoints are at the bottom of the table.

The Klepikova *et al*. (2016) data set includes heat stress data from leaf tissue. This allows for investigation of the role of stress in splice variant abundance. Heat treatment causes *SR34* to change the ratio of splice variant produced, favouring variant 2 at elevated temperatures (Lazar and Goodman, 2000). To demonstrate that this same response can be observed in the eFP‐Seq Browser, we used the Klepikova *et al*. (2016) data set, filtered the results to select for heat‐treated samples, selected variant 2 and organized from top to bottom by increasing correlation value. Visual analysis revealed that support for variant 2 does indeed increase with heat exposure. Because the Klepikova *et al*. (2016) data set features two replicates at five timepoints (1, 3, 6, 12 and 24 h), we were able to observe that the early heat treatment (1 and 3 h) had comparatively low levels of support for variant 2, the 6‐h samples had low to moderate support for variant 2 and the 12‐ and 24‐h heat‐treated samples primarily supported variant 2 (Figure 4).

**Figure 5:**
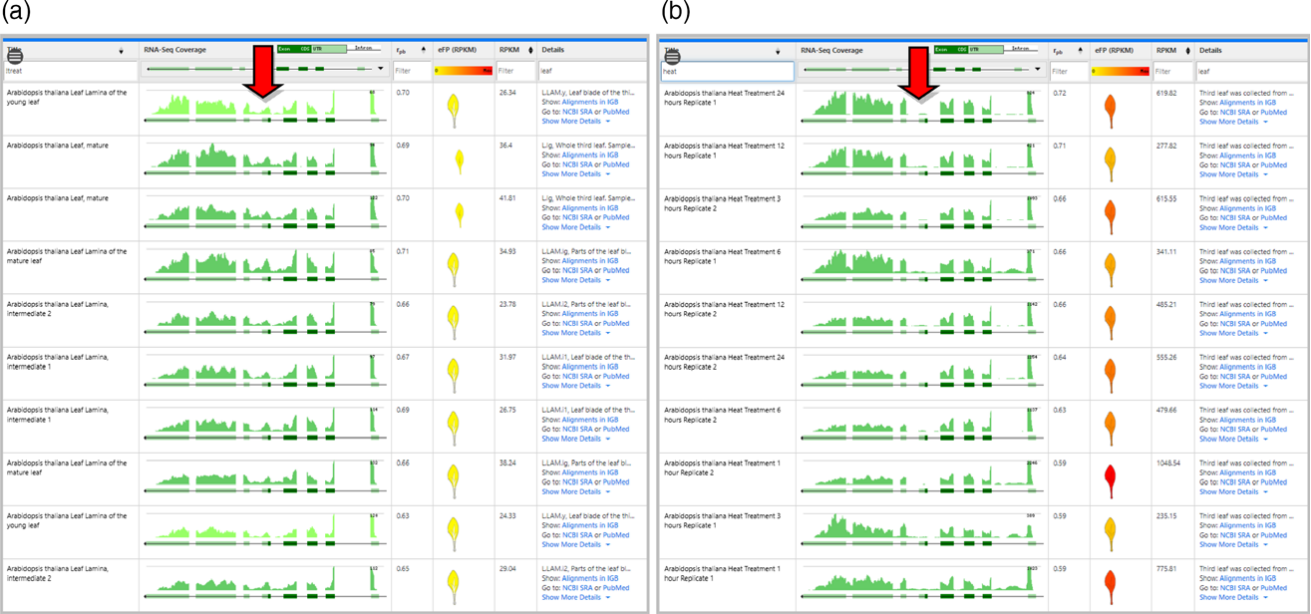
Comparison of exon skipping in *SR45a* (*At1g07350*) in untreated and heat‐treated leaf samples in the Klepikova *et al*. (2016) data set. (a) eFP‐Seq Browser results filtered for untreated leaf samples. (b) eFP‐Seq Browser results filtered for heat‐treated leaf samples. Red arrows indicate the alternatively spliced exon.

As an additional example of the stress data included as part of the Klepikova *et al*. (2016) data set, we explored the role of heat stress in exon skipping in *SR45a* (*At1g07350*). We previously found *SR45a* to have different ratios of transcripts supporting variant 1 and 2 when exposed to heat stress (Gulledge *et al*., 2012). To see if this same trend was evident within the eFP‐Seq Browser, we selected the Klepikova *et al*. (2016) data set from the dropdown menu and searched for *At1g07350*. Using the filter feature, we filtered out all of the treatment samples and selected only the leaf tissue. Visual scanning of the untreated leaf samples showed a general trend of including an alternatively spliced exon from variant 2, which leads to a premature stop codon (Figure 5a). When we instead filtered for only the heat‐treated leaf samples, we observed a much higher expression overall and a decreased proportion of support for the alternatively spliced exon, favouring the full‐length variant 1 (Figure 5b). This closely matches our previous study and shows how the eFP‐Seq Browser can be used to compare between various treatments using filtering (Gulledge *et al*., 2012).

### Using the eFP‐Seq Browser to identify new splice variants

**Figure 6:**
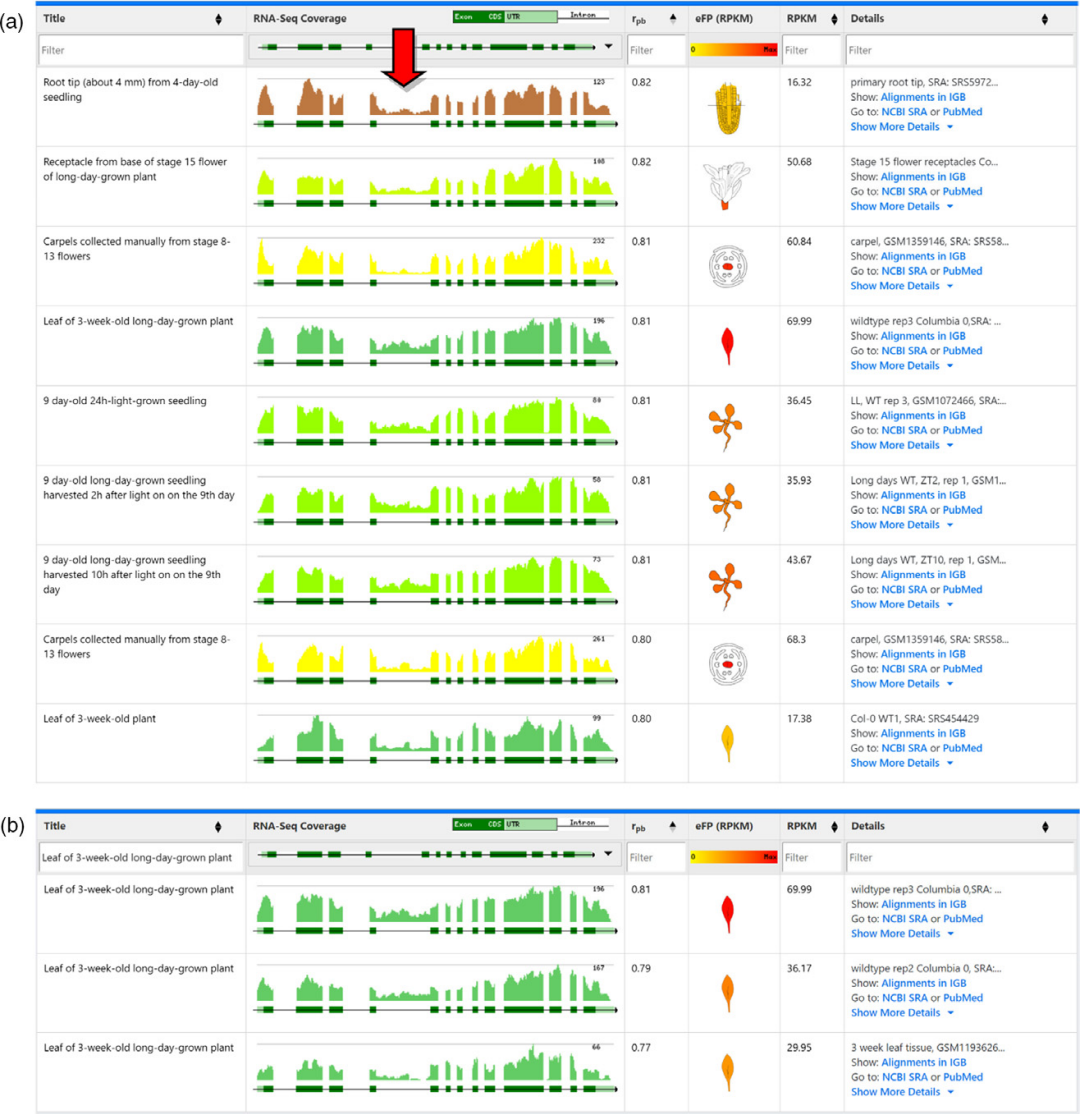
Evidence for a retained intron in *U2AF65B* (*At1g60900*) in the Araport 11 data set. (a) eFP‐Seq Browser results for *At1g60900* organized by correlation from high to low (only the top nine rows are shown). The red arrow highlights multiple tissues with reads supporting a retained fourth intron. (b) eFP‐Seq Browser results for *At1g60900* filtered by ‘Leaf of 3‐week‐old long‐day‐grown plant’ and organized by correlation from high to low. RNA‐seq samples from different laboratories support the retention event.

The eFP‐Seq Browser can also be used to visually scan for new splice variants. *U2AF65B* (*At1g60900*) is one of two genes encoding U2AF65, which helps to define 3′ splice sites. *U2AF65B* has a single splice variant in Araport. However, in our previous RNA‐seq study in 3‐week‐old leaves we found support for an intron retention event, indicating the presence of an additional splice variant (Estrada *et al*., 2015). To identify if there is any support for other splice variants in the eFP‐Seq Browser, samples were organized from top to bottom by the highest correlation value. Visually scanning through the various tissues, a number of samples showed support for a retained fourth intron (Figure 6a). Using the filter feature, we selected only the ‘Leaf of 3‐week‐old long‐day‐grown plant’ samples as this was one of the top correlation hits that had abundant reads within the possible retained fourth intron (Figure 6b). The three samples all showed evidence for the retention of the fourth intron. The data for these samples come from two separate studies, thus adding support to our own work showing evidence for the retained fourth intron (Greenberg *et al*., 2013; Bonawitz *et al*., 2014; Estrada *et al*., 2015). This illustrates the usefulness of the eFP‐Seq Browser in identifying potential new splicing events, enabling a researcher to quickly examine multiple tissues and stages across multiple studies. Again, programs like pasa (Haas *et al*., 2003) or cufflinks (Trapnell *et al*., 2010) could also be employed in this context but being able to filter samples, sort and visualize RNA‐seq coverage can be very useful too. Ultimately, potential new isoforms should be confirmed by cDNA sequencing.

## DISCUSSION AND FUTURE DIRECTIONS

**Figure 7:**
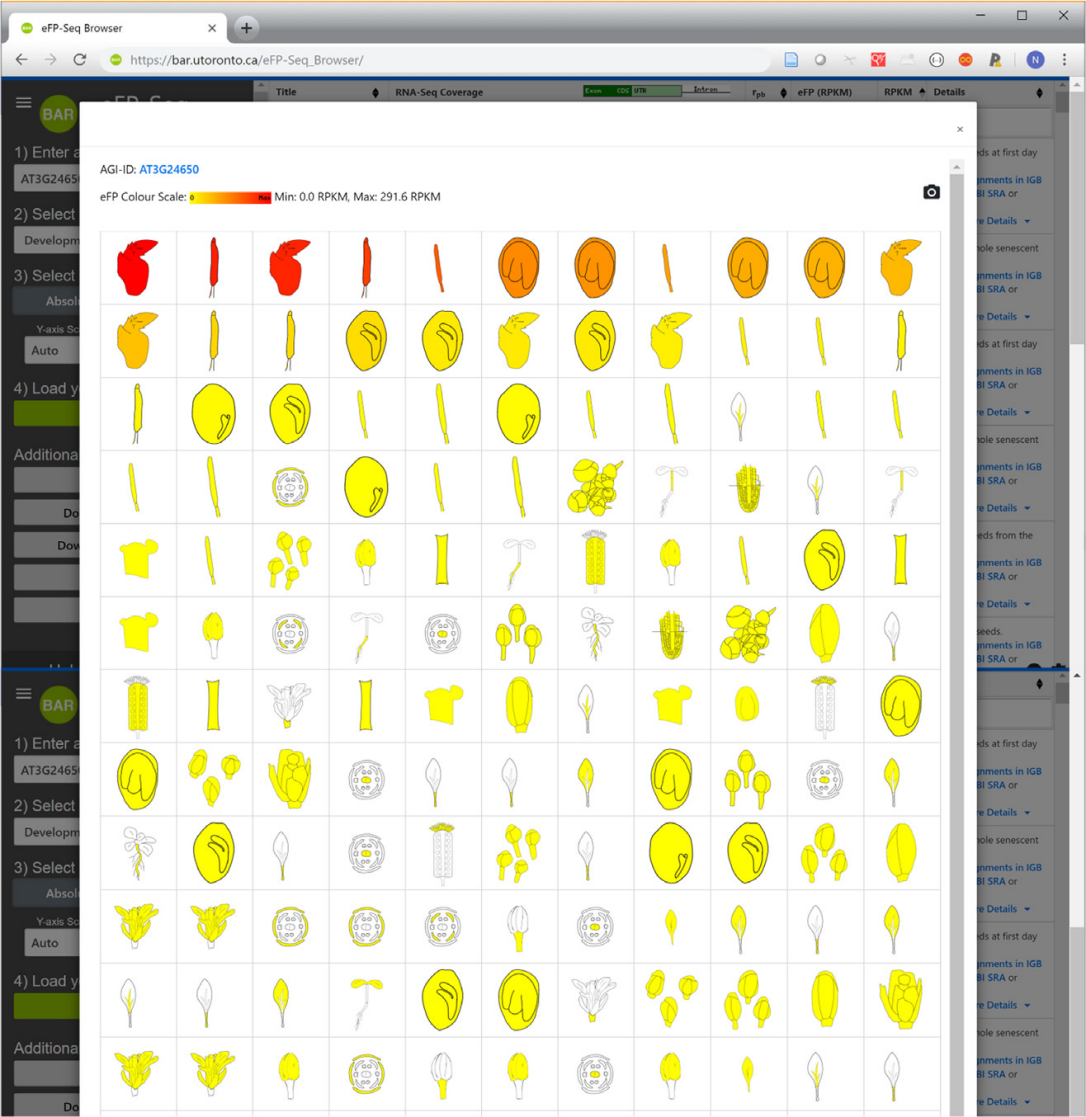
‘eFP overview’ of expression levels of ABI3 (At3g24650), sorted from strongest expression to weakest. The strongest expression is seen in whole senescent siliques (with seeds) and in seeds the first day after germination, consistent with its known biological role and with published data (e.g. Parcy *et al*., 1994).

The multi‐track eFP‐Seq Browser is an open‐source web tool for visually analysing RNA‐seq data sets. The strongest feature of this tool is its ability to calculate metrics on the fly and sort hundreds of RNA‐seq experiments by expression levels, or by congruency to a specific splice variant. The expression level comparisons are done using RPKM values, and the congruency to a specific splice variant is determined using the correlation values. The ‘eFP Overview’ feature allows for a visual overview of the expression of gene of interest in all samples in a compendium. To easily identify the samples where expression of a given gene is strongest, a researcher would sort on the RPKM column and analyse the ‘eFP Overview’ section (Figure 7).

Although each Arabidopsis RNA‐seq experiment result can be saved in a BAM file that is 1–2 GB in size and is fairly simple to handle, the combined total size of all BAM files for an organism‐wide RNA‐seq compendium can push projects like these well into the realm of biological ‘big data’. The added complexity of high data volume leads to challenges associated with big data projects. The challenges come not only from limitations of processing power but also from limitations of storage access and network transmission rate. Araport is currently hosting the 251 BAM and associated BAM index (BAI) files on Amazon's S3 data servers to offer public access. Each BCFtools mpileup call can produce an output of approximately 1–3 MB in size depending on the size of the locus. Transmitting outputs for all samples in a given expression compendium to the researcher's web browser would take a considerable amount of time because of network speed limitations and because the researcher's computer must store and work with data >100 MB in size. To mitigate this, the eFP‐Seq Browser engine performs most analyses on the BAR server and transmits only the results (mostly as compact images) to the researcher's computer.

Performing most of the analysis on the server side introduces another computational challenge because of specific shortcomings of the current version of samtools. To calculate the RPKM value for each locus in each BAM file, the number of reads mapped to the entire genomic region of the locus is required. However, this information is not part of the mpileup output even though it is used to create the mpileup. Therefore, the eFP‐Seq Browser determines the number of reads mapped to the specified genomic region separately by means of the samtools view command. This leads to the same information being extracted from the BAM files twice.

Additional coding‐related challenges include the limitations imposed by modern web browsers on the number of simultaneous HTTP requests. Currently, Google Chrome and Mozilla Firefox restrict this number to six simultaneous requests – if the server capacity allows and if this limit can be increased, it could potentially lead to a significant improvement in load time because more than six RNA‐seq read map images could be created simultaneously.

The design of the tool is such that it can be extended to include more RNA‐seq data sets by simply amending the XML configuration file with another entry. The eFP‐Seq Browser engine will automatically analyse the data set and present the new information to the researcher in the front‐end tool. Researchers wishing to view and explore their own RNA‐seq data with the eFP‐Seq Browser tool can easily do so. Researchers would make the RNA‐seq data (as BAM files) accessible on their Google Drive or Amazon S3 for instance, and then fill out a short form describing the samples, including choosing the most appropriate image to describe the sample from a library of plant part images (we have also made this library of images open access on FigShare). This information is used to generate a custom configuration file (configuration files will be stored on the BAR indefinitely unless their removal is requested by the owner). The eFP‐Seq Browser engine is able to access the BAM files and display data for a given gene based on the custom configuration file. Researchers can choose to share their data with others if desired. Furthermore, as the BAM file format is organism and sequence independent, the eFP‐Seq Browser tool can easily be extended to other organisms with minimal effort, with just two small web services based on standard GFF and Fasta files of genomic sequences needing to be modified. The eFP‐Seq Browser code is openly available on Github under a GPL v.2 licence.

## Experimental procedures

### Statistics

**Figure 8:**
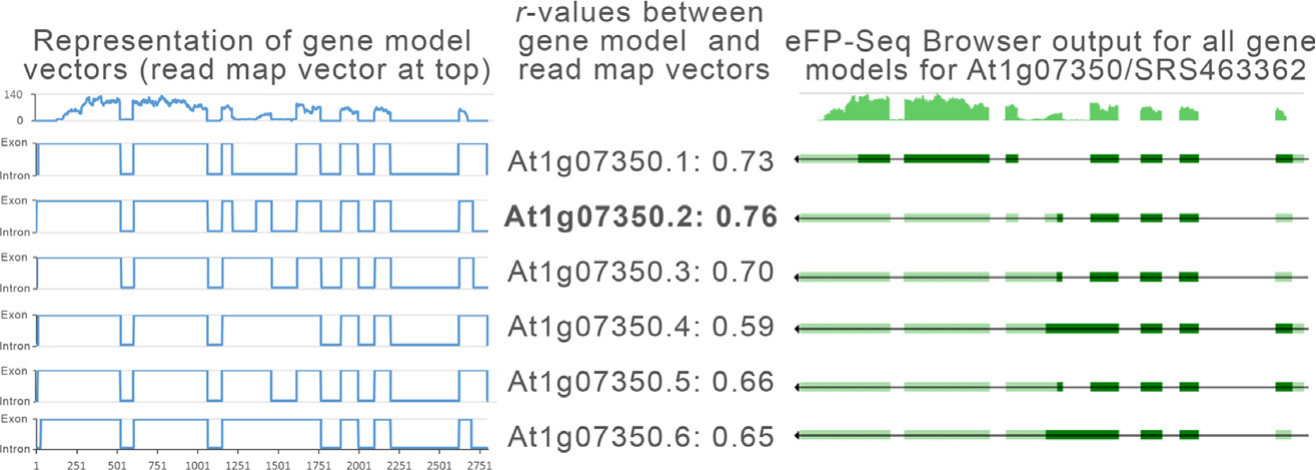
Transcript splice variants for *At1g07350* (*SR45a*) based on Araport11 genome annotation and representation of gene model vectors for these variants. The transcript splice variants for *At1g07350* (*SR45a*) based on Araport11 genome annotation (right) and representation of gene model vectors for these variants (left), along with corresponding correlation *r*_pb_‐value scores to the read map vector for data for SRA record SRS463362, calculated as described in the section ‘Statistics’. The best match is given in bold.

Two primary statistical calculations are performed by the eFP‐Seq Browser: the point biserial correlation coefficient (*r*_pb_), and the RPKM value. The correlation value is used to determine the similarity of the selected gene splice variant to the sample read coverage. A correlation *r*_pb_ value close to 1 would indicate support for a splice variant in the specific tissue/condition of the sample. The *r*_pb_ (Glass and Hopkins, 2008) allows for comparison of a vector of continuous values (i.e. RNA‐seq coverage at each nucleotide position) with one composed of dichotomous values (i.e. a nucleotide being in an exon or not). The dichotomous vector of gene model at nucleotide resolution (exon or not exon) is then compared with the number of reads mapped to each nucleotide of the gene – the coverage map. Both vectors have the same length, that being the number of nucleotides in the gene model. In practice, the ranking of *r*_pb_ values (i.e. by sorting on the *r*_pb_ column) is useful to quickly identify the sample for which the RNA‐seq coverage is closest to the selected gene model, as in Figure 8.

The purpose of the RPKM value is to enable an approximate comparison between two or more RNA‐seq experiments by standardizing the effects of the length of a gene and the size of a cDNA library in terms of the numbers of reads from it that can be mapped to the reference genome (Mortazavi *et al*., 2008). The numbers of reads mapped are standardized to the exon length of the gene (in kilobases) and to the number of reads mapped in the entire experiment (in millions of reads mapped). A gene's RPKM values in various samples can be used to identify in which tissue/condition a gene is strongly or weakly expressed. The eFP‐Seq Browser has two modes for presenting RPKM values: absolute and relative. The absolute mode displays the RPKM values for RNA‐seq data sets, using a yellow to red colour scale for the expression pictographs.

The relative RPKM mode calculates log_2_(sample RPKM over the sample controls’ average RPKM value). The relative value represents the log_2_ of the fold‐change in expression (increase or decrease) of the gene of interest in the sample relative to the sample controls (sample controls are identified in the Details element of the configuration file). A value of −2 would thus represent a four‐fold decrease relative to the control sample(s). In the relative mode, the eFP‐Seq Browser displays the expression pictographs coloured with a blue–yellow–red scale. We have curated the example data sets such that each RNA‐seq experiment has an associated control experiment or experiments used to make the relative comparison (for the Klepikova Developmental Atlas, this control sample is actually the median RPKM value across all samples). Based on the curated ‘control’ samples the average of each experiment's controls is computed, and then the log_2_ of the ratio of a given sample's RPKM value to the average of the RPKM value of the controls (or median value for cases where there is no obvious control, as for the Klepikova Atlas) is taken to be able to generate a blue–yellow–red colour scale. While statistical methods that take into account actual read abundance, such as DESeq2 (Love *et al*., 2014), should be used to call differentially expressed genes, the ‘relative’ mode of the eFP‐Seq Browser can be used to give an indication of whether a gene is induced or repressed under certain conditions, or is above or below its median expression level in the case of an expression atlas.

### samtools and data APIs

The eFP‐Seq Browser depends on two data sources: a custom RNA‐seq API based on samtools/bcftools (for data retrieval from the BAM files) and Araport's ‘gene_structure_by_locus’ API (for retrieval of gene splice variants).

The custom RNA‐seq API uses the mpileup of bcftools and view commands of samtools to extract data from the RNA‐seq data sets stored in the data layer. The mpileup command of bcftools (Li *et al*., 2009) processes data from the BAM files and returns the number of reads mapped to each nucleotide position. The view command of samtools (Li *et al*., 2009) returns all reads mapped to the specified genomic region. Combined, the two commands are used by the custom python API to extract the reads mapped to a specific region of the genome and subsequently graph the data using python to generate an image of RNA‐seq mapping coverage. This image is scaled to the highest number of reads mapped to any single nucleotide position and coloured according to the tissue of the plant it represents.

During the generation of the Araport11 genome annotation, 38.7% of the loci were found to have splice variants across different RNA‐seq experiments encompassing a variety of different tissues and treatments (Cheng *et al*., 2017). The information about the splice variants associated with each locus is retrieved using the ‘gene_structure_by_locus’ Araport web service, and a custom python API, running on the BAR server, graphs the gene structure information for the various splice variants to visually depict the splice variants. Four possible types of features are returned by the Araport gene structure web service: exon, coding sequence (CDS), 5′ untranslated region (UTR) and 3′ UTR. The UTR regions are depicted with a light green colour, while the exon/CDS features are shown by a dark green colour – to be consistent with the NCBI's colour scheme for gene structures (Figure 8, right).

In order to ensure comparability of data between samples, we recommend processing all samples using the same mapping pipeline. In the case of the Araport11 compendium, we used the BAM files generated as part of the Araport11 reannotation effort described in Cheng *et al*. (2017). In the case of the Klepikova developmental transcriptome compendium (Klepikova *et al*., 2016) we processed the short read data files from the NCBI Sequence Read Archive (project IDs PRJNA314076 and PRJNA324514) as follows: all RNA‐seq reads were processed through a quality check and trimming pipeline using fastqc (Andrews, 2010) and trimmomatic (Bolger *et al*., 2014), respectively, to remove residual adapters, low‐quality sequences (the default criteria for trimmomatic‐0.32) and reads below 36 bp. Sequences from each library were aligned to the TAIR10 genome with tophat v.2.1.1 (Kim *et al*., 2013) with bowtie2 v.2.2.8 (Langmead and Salzberg, 2012) on https://usegalaxy.org/ (Afgan *et al*., 2016) with the following custom parameters (otherwise UseGalaxy.org's default settings were used): ‐i/–min‐intron‐length 50 ‐I/–max‐intron‐length 5000.

### eFP‐Seq Browser implementation

**Figure 9:**
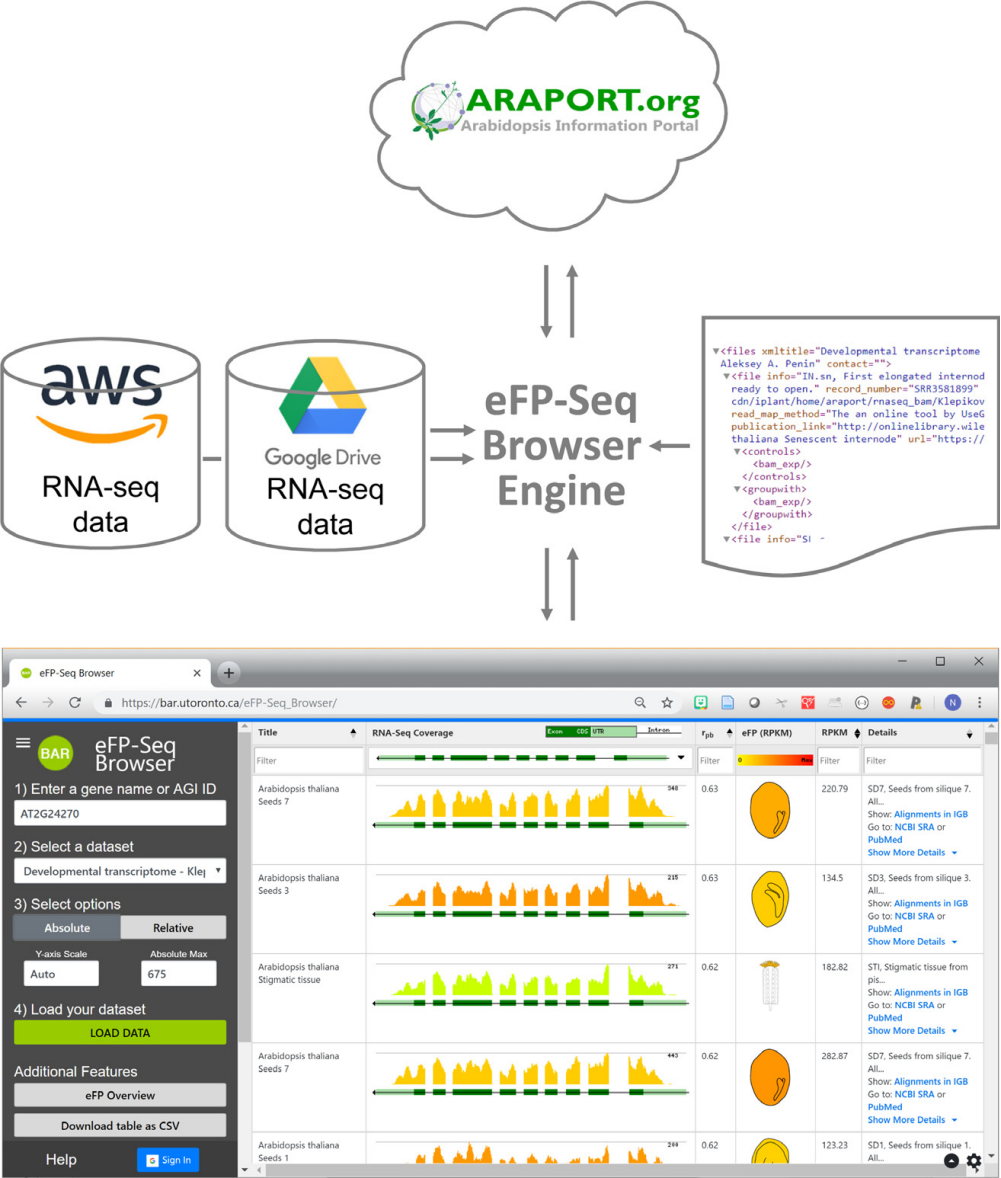
Representation of the eFP‐Seq Browser implementation. A researcher accesses the application through a web browser. The eFP‐Seq Browser engine processes the request by retrieving the appropriate information, as specified by an XML configuration file, from an API provide by https://www.araport.org/ (for gene structure information) and AWS/Google Drive (slices of BAM files corresponding to a gene's chromosomal location).

The eFP‐Seq Browser was developed as a three‐tier architecture comprising a presentation layer (front‐end web application), a logic layer (two APIs described above) and a data layer (RNA‐seq compendia or a researcher's own data, plus configuration files) (see Figure 9). The front‐end web application is written in HTML, CSS and JavaScript, and we used various open‐source libraries to speed up the development process: jQuery and jQuery UI to enable many of the functions and user interactivity features such as autocompletion for querying for AGI IDs, Bootstrap and Google's Material Design for general user interface design, ddSlick to create a dropdown to hold images, tablesorter and TableFilter to create, reorganize and query the RNA‐seq output table, and tabletoCSV to convert the RNA‐seq output table into an Excel document. The presentation and logic layers are intertwined because the front‐end JavaScript code is responsible for the point biserial correlation statistical calculations that are not possible without information from both APIs. The logic layer is described in the ‘samtools and data APIs’ section of this publication. The data layer is responsible for storing all RNA‐seq data sets that the data APIs will query. Here, it is possible to use Google Drive or Amazon S3 as a storage service. For each RNA‐seq experiment, a BAM file and a corresponding BAM Index File (BAI) file must be present for the data API to quickly extract a genomic region. The file names for each BAM repository file are stored in an extensible markup language (XML)‐based configuration file that also contains their respective titles, descriptions, SRA record numbers, total reads mapped, read map method, *Arabidopsis thaliana* plant part [scalable vector graphics (SVG) image file name and subpart if applicable], publication link, controls and replicate controls.

Using the genomic location information retrieved with the splice variants (i.e. the chromosome and the start/end positions), each of the BAM files listed in the XML file is queried to generate mapping coverage images and number of reads across the locus. This information is returned in Javascript Object Notation (JSON) format by a Python‐based API program running on the BAR server. As RNA‐seq mapping coverage information is returned, the table is updated on‐the‐fly via CSS and jQuery to show the splice variants, mapping coverage and SVG‐based eFP images for visualization of the gene's expression level in each sample. Extensive user testing was conducted to ensure the eFP‐Seq Browser was researcher‐friendly.

### Integration with the Integrated Genome Browser

The XML file described in the previous section that is used to store sample metadata is compatible with and is based on the IGB Quickload ‘annots.xml’ configuration file format (Nicol *et al*., 2009), which we extended with additional information that the eFP‐Seq Browser requires, such as which image corresponds pictographically to a given sample (e.g. a leaf image would be appropriate for a leaf sample) and the total number of reads mapped per sample. When trying to understand splicing patterns it is important to see how reads align across introns. To provide this level of detail, we implemented a link between the eFP‐Seq Browser and the IGB, a popular desktop genome browser with dozens of features designed for deep, detailed exploration of large genomic data sets, thus fulfilling Ben Shneiderman's ‘visual information seeking mantra’ for designing graphical user interfaces, namely ‘overview first, zoom and filter, then details‐on‐demand’ (Shneiderman, 1996).

To view alignments in IGB, researchers can click links labeled ‘Show: Alignments in IGB’ in the ‘Details’ column of the eFP‐Seq Browser's results table. When a researcher clicks such a link, a new web page opens at http://bioviz.org/ containing code that interprets the link, determines which data set and gene the researcher wants to see and then forwards this information to a representational state transfer (REST) endpoint within the IGB, running as a stand‐alone application on the researcher's desktop. (If IGB is not running, the page displays a message asking the researcher to download and/or start IGB and then refresh the page.) The IGB then displays the requested gene and loads the requested data set into a track, labelled with the same text shown in the eFP‐Seq Browser and using the same colour and labelling as used for the read map image. In addition, the Available Data section of IGB lists the eFP‐Seq Browser data sets inside a folder named Bio‐Analytic Resource bearing the BAR logo. Within that folder, data sets a researcher has loaded from the eFP‐Seq Browser are checked, and these are also listed in the Data Management Table in IGB. This consistency helps maintain context across the two different applications that present the same data but at different levels of detail.

### Using Google Drive for data storage

The eFP‐Seq Browser can access BAM and BAI files stored in Google Drive folders. The share link option in Google Drive can be generated and copied into eFP‐Seq Browser's ‘BAM file Repository Link’ in the ‘Generate Data’ form. A researcher would also enter other metadata to generate and download an XML file describing the researcher's own data. When this XML file is uploaded into the eFP‐Seq Browser, the Google Drive folder is mounted on the BAR server using ‘Filesystem in Userspace’ (FUSE). The Google Drive API v.3 is used to access folders and files stored in Google Drive. Custom Perl scripts build the filesystem on the BAR.

Google has a limit on the number of Google Drive API calls per minute. To overcome this, we have been granted an increase in the number of API calls per minutes after communication with Google staff. This allows us to use samtools to access data stored on the Google Drive. Data requested by samtools is transferred using HTTP ranged requests and processed by the eFP‐Seq Browser engine. An advantage of using this approach is that large BAM files do not have to be uploaded to the BAR server. Since those files are never uploaded, the researcher's data remain confidential and the BAR server never runs out of storage. A limitation of this system is the upper limit of Google Drive API calls per minute. If the limit is reached and read map graphs cannot be generated, retrying after a few minutes should solve the issue. Integration with CyVerse's Data Store is planned for the near future (Merchant *et al*., 2016).

## Author contributions

AS, PP, AP, EE, JW, AW, MC and RS built the eFP‐Seq Browser or provided resources for its construction. NHF and SRW provided examples shown in the paper. AP, MWV, CYC, APC and VK enabled AWS connectivity and provided data sets. CT, AEL and NJP wrote the manuscript with input from all authors.

## Conflict of interest

The authors declare no conflict of interest.

## Data Availability

AWS BAM file repository: https://s3.amazonaws.com/iplant-cdn/iplant/home/araport/rnaseq_bam/ and https://s3.amazonaws.com/iplant-cdn/iplant/home/araport/rnaseq_bam/Klepikova/. The SVG images are CC‐BY SA 4.0, shared on FigShare.com under the following DOIs: https://doi.org/10.6084/m9.figshare.6837926.v1 and https://doi.org/10.6084/m9.figshare.6663218.v2. eFP‐Seq Browser code is available here: https://github.com/BioAnalyticResource/eFP-Seq-Browser under a GPLv.2 licence.
